# Kolmogorov–Arnold networks for genomic tasks

**DOI:** 10.1093/bib/bbaf129

**Published:** 2025-03-31

**Authors:** Oleksandr Cherednichenko, Maria Poptsova

**Affiliations:** International Laboratory of Bioinformatics, HSE University, 11 Pokrovksy Bulvar, Moscow, 109028, Russia; International Laboratory of Bioinformatics, HSE University, 11 Pokrovksy Bulvar, Moscow, 109028, Russia

**Keywords:** Kolmogorov–Arnold networks, deep learning, large language models, diffusion models, genomic benchmarks, regulatory genomics

## Abstract

Kolmogorov–Arnold networks (KANs) emerged as a promising alternative for multilayer perceptrons (MLPs) in dense fully connected networks. Multiple attempts have been made to integrate KANs into various deep learning architectures in the domains of computer vision and natural language processing. Integrating KANs into deep learning models for genomic tasks has not been explored. Here, we tested linear KANs (LKANs) and convolutional KANs (CKANs) as a replacement for MLP in baseline deep learning architectures for classification and generation of genomic sequences. We used three genomic benchmark datasets: Genomic Benchmarks, Genome Understanding Evaluation, and Flipon Benchmark. We demonstrated that LKANs outperformed both baseline and CKANs on almost all datasets. CKANs can achieve comparable results but struggle with scaling over large number of parameters. Ablation analysis demonstrated that the number of KAN layers correlates with the model performance. Overall, linear KANs show promising results in improving the performance of deep learning models with relatively small number of parameters. Unleashing KAN potential in different state-of-the-art deep learning architectures currently used in genomics requires further research.

## Introduction

Deep learning models have been successfully applied to a wide range of genomic tasks including prediction of variant effect [1, 2], and location of functional genomic elements, such as promoters [3], enhancers [4], transcription factor binding sites [5], histone marks [6, 7], flipons [8, 9], splice sites [10], and others. Neural networks also proved to be efficient in the field of RNA biology (see [11] and [12] for review) predicting RNA-protein binding [13], RNA structure [14], and noncoding RNA [15]. The evolution of deep learning applications in genomics followed the path of the evolution of deep learning architectures. It started with pioneering application of convolutional neural networks (CNNs) [1, 16] and reccurent neural networks (RNNs) to DNA sequences [17], and then continued with Transformer-based large language models (LLMs) such as DNABERT [18], DNABERT2 [19], and Nucleotide Transformer [20]. DNABERT was trained on one reference human genome, while DNABERT2 and Nucleotide transformer were trained on the multi-species genome comprising up to 40B of bases pairs. After training, foundation models are fine-tuned on different downstream tasks. However, even with parameter efficient fine-tuning techniques, resource limitations can impose significant constraints on the task execution.

Transformers require substantial computational resources due to quadratic attention scaling, and the next architecture Hyena, based on long convolutions, incorporated longer context up to 1M nucleotides by scaling subquadratically, which resulted in HyenaDNA [21]. Another promising alternative to overcome transformers’ computational inefficiency is Mamba architecture [22], which lies at the basis of Caduceus model in genomics [23]. Both Hyena and Mamba achieved a certain tradeoff between computational resources and performance quality. In comparison with the latest genomic foundational model DNABERT2, HyenaDNA has 10x less parameters and on some genomic tasks outperforms DNABERT2.

With an evergrowing size of deep learning architectures, it has been a challenge to find smaller architectures that would perform equally well. Here, we aim to explore the potential of small neural networks that utilize only few multilayer perceptrons (MLPs), incorporating recent innovations in deep learning, such as Kolmogorov–Arnold networks (KANs) [24].

The neural networks discussed above have one thing in common: they rely on the universal approximation theorem. One of the recent advances in deep learning architectures, KAN [24], leverages the Kolmogorov–Arnold theorem to incorporate splines into a neural network architecture, offering a compelling alternative to traditional MLPs. KANs have already been successfully applied in different areas such as mechanics [25, 26], computer vision [27–31], NLP [32, 33], time series [33–35], physics [24, 25, 36], speech enhancement [37], molecular representations [38, 39], and recommendation systems [40]. Inspired by this advancement, multiple modifications have emerged that attempt to overcome various issues associated with the spline-based approach, namely computational overhead and a large number of trainable parameters.

In this study we aim at evaluating the potential of KAN-based models in genomics. Given the limitations in computational resources, we applied KAN to simple convolutions and dense networks with a relatively small number of parameters. We tested KANs to the task of classification and generation by replacing layers in the baseline model with linear KAN layers (LKAN) and convolutional KAN (CKAN) layers. The test was performed on three benchmark datasets: Genomic Benchmarks [41], Genome Understanding Evaluation (GUE), and our collection of datasets for flipons or non-B DNA structures [42].

### Kolmogorov–Arnold networks

#### Linear Kolmogorov–Arnold networks

Here we briefly introduce KAN and present key features of this architecture. MLPs [43] are inspired by the universal approximation theorem that states that a feed-forward network with a single hidden layer containing a finite number of neurons can approximate continuous functions on compact subsets of $\mathbb{R}^{d}$. KAN [44] focuses on the Kolmogorov–Arnold representation theorem [45], which states that any multivariate continuous function can be represented as a composition of univariate functions and the addition operation:


(1) \begin{align*}& f(\mathbf{x})=\sum_{k=1}^{2n+1}\Phi_{k} \left ( \sum_{l=1}^{n}\phi_{k,l}(x_{l}) \right ),\end{align*}

where $\phi _{k,l}$ are univariate functions that map each input variable $x_{l}$ as follows: $\phi _{k,l}:[0,1] \to \mathbb{R}$. The authors of the original KAN paper proposed how to extend the KAN application to deep learning networks by stacking KAN layers.


(2) \begin{align*}& f(\mathbf{x})=\sum_{i_{L-1}=1}^{n_{L-1}}\phi_{L-1, i_{L}, i_{L-1}} \left (\sum_{i_{L-2}=1}^{n_{L-2}} \left ( \sum_{i_{0}=1}^{n_{0}} \phi_{0,i_{1}, i_{0}}(x_{i_{0}}) \right ) \right )\end{align*}

Similarly it is possible to rewrite it in the following way:


(3) \begin{align*}& \mathrm{KAN}(\mathbf{x})=(\Phi_{L-1} \circ... \circ \Phi_{1} \circ \Phi_{0})\mathbf{x}\end{align*}

Implementation of KANs is provided by the authors of [24]; however there are several aspects that could limit the scalability of KANs: a large number of trainable parameters, training time, and inference time. On top of that, in [32] the authors claim that weights have different initialization that makes it impossible to keep variance-preserving initialization [29]. To overcome these issues, in [29] the authors suggest an efficient implementation of KANs with the straightforward matrix multiplication and L1 regularization for the model’s weights.

#### Kolmogorov–Arnold convolutions

Kolmogorov–Arnold convolutions are proposed in [27] and designed to be similar to CNNs. The difference is that the convolutional layers are replaced by KAN convolutional layers, and, after flattening, one can use either KAN or MLP. The advantage of the CKANs is that they have significantly fewer parameters compared with other architectures. This is provided by the network architecture, because B-Splines are capable of smooth representations of arbitrary activation functions, which cannot be determined with ReLU. The authors concluded that Kolmogorov–Arnold convolutions are capable of achieving high performance as compared with original convolutional networks [28].

### Genomics tasks

#### DNA classification

Classification of DNA sequences is one of the most common tasks in genomics, which is solved with deep learning models. In this work, we perform classification on three large benchmark datasets: Genomic Benchmarks [41], Genomic Understanding Evaluation [19], and Flipon Benchmark assembled by us. For each of the datasets a train and a test sets are available.

#### DNA generation

Another important application of deep learning in genomics is generative modeling. Generative models are usually used in data augmentation approaches to generate new samples from the learned distribution.

There are many types of generative models, but here we focus on the most popular models for DNA sequence generation: denoising diffusion implicit model (DDIM) [46] and generative adversarial network (GAN) [47]. In generative approaches, first, a generative model is trained on a train set, and the trained model produces synthetic samples of the same size as the test set. Secondly, synthetic data are supplied to a classifier trained on the real data to evaluate how good are generative models in capturing main patterns of the real data.

We will use the Flipons collection [48–53] for this task (see Methods).

## Methods

### Models

**Figure 1:**
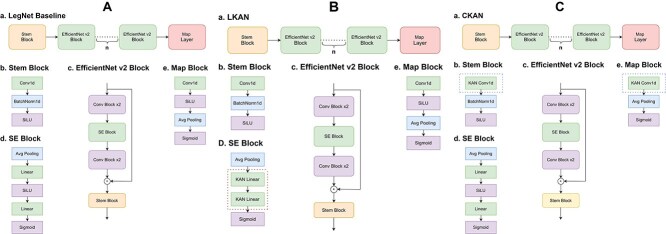
**A. LegNet-based Baseline. a**. Architecture of Baseline LegNet-based convolutional neural network. $n$$n=6$  **b**. Stem block. **c**. Main convolutional EfficientNet-like [55] block. **d**. Mid SE block with bilinear layer. **e**. Final block. **B. LegNet-based LKAN. a**. Architecture of Baseline LegNet-based convolutional neural network with LKAN. **b**. Stem block. **c**. Main convolutional EfficientNet-like [55] block. **d**. Mid SE block with replaced LKAN layers. **e**. Final block. **C. LegNet-based CKAN. a**. Architecture of Baseline LegNet-based convolutional neural network with CKAN. **b**. Stem block with replaced CKAN layer. **c**. Main convolutional EfficientNet-like [55] block. **d**. Mid SE block. **e**. Final block with replaced CKAN layer.

We use pytorch efficient implementation of KAN (EKAN) [29] as linear KAN layers. We use KAN-Conv [27] implementation as KAN convolutional neural network. We incorporate KAN layers into convolutional neural network that takes its core from Leg-Net [54]. We compare the impact of KAN layers by replacing MLP modules with KANs (see Tables 1–5). To make a comparison more fair and to exclude model size effect, in all the experiments for classification we used models of equal size: 22M parameters. We used the following notation for our tested models (Fig. 1): Baseline for LegNet-based convolutional neural network, LKAN for LegNet-based convolutional neural network [55] with incorporated Linear KANs, and CKAN for LegNet-based convolutional neural network with incorporated Convolutional KANs. Since CKANs scales with larger grid size (see Results), combining CKAN and LKAN in our architecture was impossible due to limitations in resources, thus hybrid CKAN-LKAN architectures were not conducted.

**Figure 2:**
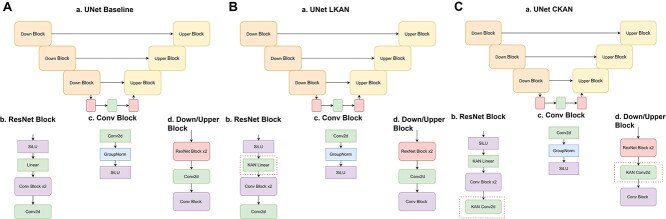
**A. Unet-based Baseline. a**. Architecture of Baseline DNA-Diffusion Unet-based model. **b**. Base ResNet block. **c**. Convolutional Block at the end of upper and down blocks. **d**. Down and upper blocks with ResNet blocks. **B. Unet-based LKAN. a**. Architecture of LKAN modification of DNA-Diffusion Unet-based model. **b**. Base ResNet block where dense layer is replaced with LKAN. **c**. Convolutional block at the end of upper and down blocks. **d**. Down and upper blocks with ResNet blocks. **C. Unet-based CKAN. a**. Architecture of CKAN modification of DNA-Diffusion Unet-based model. **b**. ResNet block where convolutional blocks are replaced with CKANs. **c**. Convolutional block at the end of upper and down blocks. **d**. Down and upper block with ResNet block where convolutional layer is replaced with CKAN.

In experiments with DNA sequence generation we utilized DDIM [46] designed specifically for DNA [57]. We replaced linear layers with KAN layers in Unet like architecture (Fig. 2).

**Figure 3:**
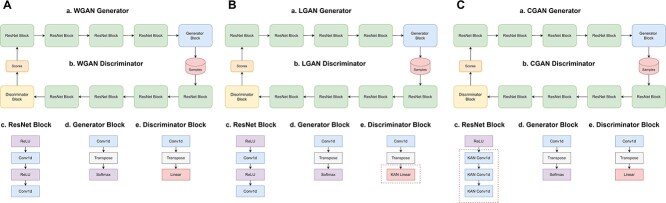
**A. Wasserstein GAN Baseline. a**. ResNet-based WGAN Generator. **b**. ResNet-based WGAN Discriminator. **c**. ResNet block. **d**. Generator block. **e**. Discriminator block. **B. Wasserstein GAN with LKAN. a**. ResNet-based LGAN Generator. **b**. LGAN Discriminator where MLP is replaced with LKAN. **c**. Base ResNet block. **d**. Generator block. **e**. Discriminator block with LKAN. **C. Wasserstein GAN with CKAN. a**. ResNet-based CGAN Generator **b**. ResNet-based CGAN Discriminator. **c**. ResNet block with CKAN layers. **d**. Generator block. **e**. Discriminator block.

We used a Wasserstein GAN [47] as another commonly used architecture to produce synthetic DNA sequences. Also, we replaced MLP block with KAN layers to create LGAN and CGAN (Fig. 3).

### Datasets

#### Genomic benchmarks

We use the following notations for all datasets: DMEE—dummy mouse enhancers ensemble, DCIS—demo coding vs intergenomic seqs, DHW—demo human or worm, DES—drosophila enhancers stark, HEC—human enhancers cohn, HEE—human enhancers ensembl, HER—human ensembl regulatory, HNP—human nontata promoters, HOE—human ocr ensembl.

#### Flipons, or non-B DNA structures

Inspired by Genomic Benchmarks we created a collection of datasets for detecting flipons [42], or non-B DNA structures: Z-flipons, or Z-DNA [48, 49]; G-flipons, or G-quadruplexes (GQs) [50–53]; H-flipons, or H-DNA (triplexes) [48]. We provide train and test sets trying to prevent data leakage. Full description of datasets are available in Supplementary Table 1. Similar to Genomic Benchmarks datasets we use the following notations for all flipons datasets: ENDO—Endoquad GQs; G4seq—G4-seq experimental dataset for GQs; G4ChIP—G4 ChIP-seq experimental data set for GQs; G4cut—G4 CUT&Tag experimental dataset for GQs; ZKOU—Kouzine et al. experimental dataset for Z-DNA; ZShin—Shin et al. ChIP-seq experimental dataset for Z-DNA; HKou—Kouzine experimental dataset for H-DNA.

#### Genome understanding evaluation

We follow [19] authors in evaluation on Genome Understanding Evaluation (GUE) and extended version (GUE+) to study models’ performance on a wider range of genomic tasks: prediction of promoter, core promoter, splice sites, epigenetic marks, transcription factor (TF) binding sites on human and mouse, and covid variant classification.

## Results

### Benchmarks on DNA classification

#### Genomic benchmarks

KAN performance in comparison with the baseline model for nine datasets from Genomic Benchmarks [41] are presented in Table 1 (MCC score averaged from five-folds). Baseline CNN model is of equal size with LKAN and CKAN. Other classification metrics such as accuracy, ROC-AUC, precision, recall, and F1 are provided in Supplementary Tables 2–14. We observe that both LKAN and CKAN improve the quality of the model, yet CKAN requires higher value of grid size parameter.

**Table 1:** KAN performance on classification task for genomic benchmarks based on MCC-score averaged over five-folds

Data set	Baseline	LKAN	CKAN
DMEE	*70.2 $\pm $ 0.5*	**73.2 $\pm $ 0.2**	64.8 $\pm $ 1.3
DCIS	81.1 $\pm $ 0.5	**85.5 $\pm $ 1.4**	*81.7 $\pm $ 1.2*
DHW	*89.2 $\pm $ 0.5*	**90.2 $\pm $ 0.5**	88.1 $\pm $ 0.8
DES	**27.3 $\pm $ 0.9**	*28.9 $\pm $ 0.6*	25.3 $\pm $ 0.6
HEC	66.6 $\pm $ 0.8	**68.2 $\pm $ 0.5**	*67.9 $\pm $ 0.8*
HEE	*63.2 $\pm $ 0.5*	**69.9 $\pm $ 0.5**	59.0 $\pm $ 1.0
HER	**90.0 $\pm $ 0.8**	*89.3 $\pm $ 0.8*	88.4 $\pm $ 0.6
HNP	*87.9 $\pm $ 0.9*	**88.8 $\pm $ 0.9**	85.0 $\pm $ 1.0
HOE	*70.8 $\pm $ 0.6*	**74.4 $\pm $ 0.7**	68.9 $\pm $ 1.0

The best values are in **bold**, and the second best values are in *italics*.

#### Flipons, or non-B DNA structures

KAN’s performance on seven datasets from Flipon collection [48–53] is presented in Table 2. Baseline CNN model is of equal size with LKAN and CKAN.

**Table 2:** KAN performance on classification task for flipons based on MCC-score averaged over five-folds

Data set	Baseline	LKAN	CKAN
ENDO	*89.1 $\pm $ 0.5*	**93.5 $\pm $ 0.4**	89.0 $\pm $ 0.8
G4SEQ	*83.3 $\pm $ 0.6*	**89.0 $\pm $ 0.8**	84.5 $\pm $ 0.2
G4CHIP	90.2 $\pm $ 0.9	**92.2 $\pm $ 0.7**	*91.3 $\pm $ 1.5*
G4CUT	*91.1 $\pm $ 0.8*	**94.8 $\pm $ 0.7**	85.6 $\pm $ 1.2
ZKOU	*94.5 $\pm $ 1.0*	**96.3 $\pm $ 0.8**	94.0 $\pm $ 0.8
ZSHIN	**97.9 $\pm $ 0.5**	*97.0 $\pm $ 0.5*	95.7 $\pm $ 1.1
HKOU	*90.5 $\pm $ 0.6*	**95.2 $\pm $ 1.1**	88.7 $\pm $ 0.9

The best values are in **bold**, and the second best values are in *italics*.

#### Genome understanding evaluation

Results of KAN’s performance in comparison with the baseline model for nine datasets from Genome Understanding Evaluation [19] are presented in Table 3 (MCC score averaged from five-folds). Baseline CNN model is of equal size with LKAN and CKAN.

**Table 3:** KAN performance on classification task for GUE based on MCC averaged over five-folds

Data set	Baseline	LKAN	CKAN
H3	*70.7 $\pm $ 1.5*	**71.3 $\pm $ 1.0**	69.4 $\pm $ 1.5
H3K14AC	40.2 $\pm $ 0.8	**43.2 $\pm $ 0.7**	*41.0 $\pm $ 1.0*
H3K36ME3	*44.3 $\pm $ 0.9*	**47.7 $\pm $ 0.6**	42.9 $\pm $ 0.9
H3K4ME1	35.6 $\pm $ 0.4	**40.0 $\pm $ 0.8**	*35.8 $\pm $ 1.1*
H3K4ME2	*27.2 $\pm $ 0.5*	**29.3 $\pm $ 0.5**	24.3 $\pm $ 1.2
H3K4ME3	*28.4 $\pm $ 0.5*	**29.8 $\pm $ 0.4**	27.2 $\pm $ 0.7
H3K79ME3	60.8 $\pm $ 0.9	**62.1 $\pm $ 0.8**	*61.1 $\pm $ 1.5*
H3K9AC	*50.5 $\pm $ 0.6*	**51.9 $\pm $ 0.5**	50.0 $\pm $ 0.9
H4	*75.6 $\pm $ 0.9*	**77.2 $\pm $ 0.7**	70.9 $\pm $ 0.8
H4AC	33.9 $\pm $ 0.8	**38.1 $\pm $ 0.8**	*36.2 $\pm $ 0.9*
PD ALL	75.9 $\pm $ 1.1	**81.2 $\pm $ 1.4**	*77.2 $\pm $ 1.2*
PD NOTATA	*88.2 $\pm $ 1.1*	**89.9 $\pm $ 0.7**	87.1 $\pm $ 1.2
PD TATA	56.1 $\pm $ 0.5	**58.2 $\pm $ 0.8**	*57.9 $\pm $ 0.9*
TF HUMAN 1	*69.9 $\pm $ 1.0*	**72.4 $\pm $ 0.8**	66.3 $\pm $ 1.3
TF HUMAN 2	*49.6 $\pm $ 0.4*	**54.3 $\pm $ 0.7**	47.2 $\pm $ 0.7
TF HUMAN 3	43.2 $\pm $ 0.5	**46.8 $\pm $ 0.5**	*44.4 $\pm $ 0.9*
TF HUMAN 4	*72.6 $\pm $ 0.8*	**72.9 $\pm $ 0.5**	71.3 $\pm $ 0.9
TF HUMAN 0	65.1 $\pm $ 0.7	**67.4 $\pm $ 0.6**	*65.5 $\pm $ 0.9*
CPD ALL	64.8 $\pm $ 0.9	**68.2 $\pm $ 0.6**	*66.2 $\pm $ 1.1*
CPD NOTATA	*64.5 $\pm $ 0.7*	**67.2 $\pm $ 0.8**	63.1 $\pm $ 1.1
CPD TATA	72.3 $\pm $ 0.9	**78.2 $\pm $ 0.9**	*74.7 $\pm $ 1.2*
TF MOUSE 1	*81.0 $\pm $ 0.7*	**82.8 $\pm $ 0.6**	80.1 $\pm $ 0.8
TF MOUSE 2	79.8 $\pm $ 0.6	**85.5 $\pm $ 0.8**	*81.2 $\pm $ 0.7*
TF MOUSE 3	77.6 $\pm $ 1.2	**81.4 $\pm $ 0.7**	*79.2 $\pm $ 0.9*
TF MOUSE 4	*42.6 $\pm $ 0.7*	**48.7 $\pm $ 0.5**	40.1 $\pm $ 0.7
TF MOUSE 0	38.2 $\pm $ 0.7	**40.1 $\pm $ 0.6**	*39.0 $\pm $ 0.9*
Virus	50.3 $\pm $ 0.9	**58.2 $\pm $ 1.5**	*50.5 $\pm $ 0.6*
Splice	*78.2 $\pm $ 0.8*	**80.0 $\pm $ 0.8**	75.2 $\pm $ 0.7

The best values are in **bold**, and the second best values are in *italics*.

We can see that in almost all classification tasks LKAN outperforms Baseline and CKAN models on average by 7.71% and 9.59%, respectively. The largest difference was detected for virus classification with 70.53% increase over baseline and 59.73% increase over CKAN, but this is because the baseline model does not perform well having only an MCC of 20.7.

Increase by 16%–22% in LKAN over Baseline and 16%–30% in LKAN over CKAN was reached for enhancer–promoter interaction datasets, and here too the baseline models do not perform well and have an MCC range of 34.3–38.2. For histone marks we observe a larger difference in performance for those genomic elements that are not predicted well by baseline models: these are H3K4ME1 (12.36% for LKAN over Baseline with MCC of 35.6) and to a lesser extent H3K4ME2 (8% for Baseline with MCC of 27.2) and H3K4ME3 (5% for LKAN over Baseline with MCC of 28.4). For human TFs, the biggest difference is for PAX5 (9%) and TRIM28 (8%), which are not well-predicted by baseline (MCC 49.6 and 43.2 correspondingly). The same trend is observed for mouse TFs with the largest increase of 14% for Nelfe, which has a low MCC of 42.6 for baseline. For high MCC baseline models the increase is small such as for human H3 (0.85%) with the Baseline MCC of 70.7 and MXI1 (0.41%) with the Baseline MCC of 72.6. For flipon dataset, an increase in performance is also small in 2%–6% for LKAN over Baseline and 1%-11% for LKAN over CKAN but the Baseline model range is also high 83.3-97.9.

As for CKAN performance, on many genomic datasets CKAN showed up the second-best after LKAN, and this was the case for different classes of genomic elements such as histone marks, TFs, promoter–enhancer interactions, flipons. Here we think it is early to make a conclusion because incorporation of CKAN layers is not a trivial task and still a subject of active discussion (see Discussion), but our results point to the promising usage of CKANs along with LKANs too.

In general we can see the larger increase in performance for LKAN for less structured data, i.e. functional genomic elements lacking a clear DNA sequence motif or regular pattern, such as enhancer–promoter interactions, or widely distributed histone marks, or TF binding sites without a well-defined DNA motif. This suggests that LKAN’s architecture is particularly effective for more challenging genomic tasks. In contrast, for data with strong sequence patterns—such as the Z-DNA in the Kouzine dataset, which exhibits an alternating purine-pyrimidine pattern—the baseline MCC of 94.5 is already quite high; consequently, the increase to 96.3 with LKAN is less pronounced.

**Table 4:** KAN performance on classification task for GUE+ based on MCC averaged over five-folds

Data set	Baseline	LKAN	CKAN
GM12878	36.6 $\pm $ 1.1	**41.2 $\pm $ 0.9**	*37.0 $\pm $ 1.2*
HELA-S3	*34.3 $\pm $ 1.0*	**40.8 $\pm $ 1.2**	31.3 $\pm $ 0.9
HUVEC	*37.2 $\pm $ 0.7*	**39.6 $\pm $ 0.9**	30.2 $\pm $ 0.8
IMR90	*34.5 $\pm $ 0.9*	**42.0 $\pm $ 0.9**	33.3 $\pm $ 1.3
K692	*38.2 $\pm $ 0.9*	**44.4 $\pm $ 0.7**	37.9 $\pm $ 1.1
NHEK	*33.7 $\pm $ 1.1*	**40.1 $\pm $ 0.9**	34.4 $\pm $ 0.6
FUNGI	*51.1 $\pm $ 0.6*	**55.5 $\pm $ 1.0**	50.4 $\pm $ 1.2
VIRUS	*20.7 $\pm $ 0.5*	**35.3 $\pm $ 1.9**	22.1 $\pm $ 1.8

The best values are in **bold**, and the second best values are in *italics*.

Considering results for the classification tasks we observe that Linear KAN scales better than convolutional KAN of the same size. We present visualization of averaged model’s performance for models of various size in Fig. 3. Additionally, we provide results of experiments on how grid size parameter affects the performance of LKAN and the number of total parameters along with the time per batch training. We evaluated these results on GUE datasets.

### Benchmarks on DNA generative models

To test KAN’s capability in producing synthetic DNA sequences we incorporated Linear KAN layers into denoising Unet from DNA Diffusion framework [57]. We compared final loss for various models (Table 5) and observed that LKAN is capable of reaching the lowest validation loss given equal amount of parameters with baseline Unet. CKAN requires x2 parameters to achieve the comparable metrics.

**Table 5:** KANs for DNA generation with DNA-Diffusion: KAN loss (SP-MSE) comparison on flipon generation

DNA-Diffusion			
Task	Unet (410M)	LKAN (440M)	CKAN (820M)
G4	*0.0334*	**0.0276**	0.0340
ZDNA	0.0301	**0.0222**	*0.0299*
HDNA	0.0388	**0.0281**	*0.0379*

The best values are in **bold**, and the second best values are in *italics*.

Additionally, we observe that KAN affects training stability of Unet, as it is presented in Fig. 5. Both trainings utilize exponential moving average (EMA) for smoother performance. We conclude that LKANs provide better performance than CKANs, yet CKANs require higher values for grid size, which significantly makes the model heavy in terms of parameters. For instance, CKAN requires 820M parameters, while LKAN requires 440M to outperform baseline Unet.

On top of that, we tested KAN by adding it into architecture of Wasserstein GAN [47]. WGAN architecture is composed of a generator and a discriminator (see Fig. 3), and each of these networks consists of five ResNet blocks. Each block consists of a convolutional layer with 5x5 kernel and padding 2, followed by ReLU activation. Training of all GAN models - WGAN, LGAN, CGAN - was accompanied by calculation of two edit distances [58]: $D_{self}$ is the average edit distance in a set of 1000 generated sequences where distance is measured between the sequence and its nearest neighbor except for itself (Table 6). This metric is also measured in the test and training datasets (Fig. 6). If the generated data are as diverse as the data in the training and test datasets, the values of $D_{self}$ in the generated sequences will be similar. $D_{train}$ is the average distance that is used to measure GAN training. The low value of this metric alerts the overfitting of the GAN on the training set.

We compare the performance of KAN in generative design by evaluating Kullback–Leibler (KL) divergence and Wasserstein distance (WD) (Fig. 7 and 8). Since diffusion model requires substantial resources for training and inference (see Supplementary Materials for details), we conduct these experiments only for flipon’s datasets [58]. We compare the diversity calculated on edit distance within a sample of synthetic sequences for various models (Table 7). Higher values ensure more diversity within a sample. Trained generative models have captured the prior distributions of flipons based on calculated distances between real and generated distributions.

We observe that diversity is relatively the same for all types of models. Incorporation of linear and convolutional layers into DNA-Diffusion framework, a large U-net-like model with ResNet convolutional blocks, might not be the core factor influencing generation of diverse synthetic sequences. In all experiments LKAN outperforms both CKAN and Baseline by a very small margin (Table 7). We also note that the absolute values of diversity of real sequences in our datasets are also around 72 depending on different flipons [58]. And here for the generation task CKAN can be more efficient than LKAN if generated sequences have an underlying pattern as in the case with quadruplexes, though the overall difference between LKAN and CKAN in performance is <1%. Here we conclude that current implementation of KAN layers does not significantly affect the diversity of synthetic samples.

**Table 6:** KANs for DNA generation with Wasserstein GAN: comparison of edit distance and wasserstein cost on flipon generation

Wasserstein GAN			
Task	WGAN (20M)	LGAN (20M)	CGAN (30M)
G4	*250*	**252**	249
ZDNA	*223*	**225**	220
HDNA	*253*	**255**	250

The best values are in **bold**, and the second best values are in *italics*.

**Table 7:** KANs for DNA generation: metrics of diversity for flipons

DNA-Diffusion			
Task	Unet (410M)	LKAN (440M)	CKAN (820M)
G4	69.2	*70.3*	**73.2**
ZDNA	70.4	**72.2**	*71.1*
HDNA	64.8	**64.9**	64.8
**Wasserstein GAN**			
**Task**	**Unet (20M)**	**LKAN (20M)**	**CGAN (30M)**
G4	68.9	**71.0**	*70.7*
ZDNA	70.5	**71.9**	*71.2*
HDNA	63.6	**64.7**	*64.4*

The best values are in **bold**, and the second best values are in *italics*.

### Ablation study

Additionally, we investigated the impact of the total amount of replaced MLP layers with KANs. For this experiment we used human enhancers cohn dataset from genomic benchmarks collection. Taking into consideration the total number of EfficientNet-like blocks (Supplementary Table 16) we decided to determine if the same quality can be achieved by replacing only few blocks with LKANs. Model performance based on F1 score with the number of replaced MLP layers and the grid size is presented in Supplementary Table 16. We observe a steady increase in performance with the number of replaced layers. We also see that increasing grid size positively impacts the overall performance. Thus, we found that the best performance is achieved when all blocks are replaced with LKANs; however, with higher grid size values it is possible to achieve fair performance with $N=4$ or $N=5$ (see Supplementary Tables 16 and 17). Yet, increasing grid size makes model heavier, therefore the training process takes longer. Moreover, higher grid size is prone to certain overfit, when training metrics are close to high values, while testing metrics remain constant (Supplementary Figure 1).

## Discussions and conclusions

In this study, we evaluated performance of the recently developed KANs in the domain of genomics. For that we used a wide range of datasets to cover different types of genomic functional elements including promoters, enhancers, histone marks, and others collected in two published genomic benchmark datasets and one novel benchmark dataset for flipons, or non-B DNA structures, assembled by us. For our baseline models i.e. models without KAN, we utilized LegNet-based convolutional network for DNA classification [54], Wasserstein GAN [47], and DDIM DNA-Diffusion with a core Unet module for generating synthetic sequences [57]. MLP and convolutional layers in these baselines were replaced with linear and convolution KAN layers correspondingly.

Our benchmarking shows that LKANs show promising results in replacing MLPs in a broad range of genomic tasks, while CKANs struggle with scaling over large number of parameters, which require more computational resources. In sequence generation we proved that all models, baseline and KANs, can learn distributions of the input data (Figs 5 and 6); however LKAN outperforms baseline and CKAN in terms of loss, while CKAN requires x2 more parameters to achieve the same performance as LKAN. In diversity of generated samples both LKANs and CKANs outperform baseline but the difference is <1%. To enhance diversity in generative tasks, the learning paradigm (i.e diffusion process or adversarial training) plays a major role.

In the study published as preprint [59] the authors utilize the original vanilla version of KAN [24] as the final layer of their models, which are hybrid CNN, RNN, and attention networks named CRA-KAN. CRA-KAN outperforms state-of-the-art models on 50 ChIP-seq datasets, which is in line with the results we presented using another implementation of LKAN [27].

In the original KAN paper [24] the authors demonstrated that KANs improve in accuracy over MLPs on small-scale tasks. We showed that improvement is higher for less structured genomic data. Implementation of B-splines [25] is more technically accurate than vanilla MLP approximation approach. In addition, LKAN utilizes several advanced techniques such as efficient initialization of the spline scaler and weights regularization that can also contribute to LKAN superior performance.

**Figure 4:**
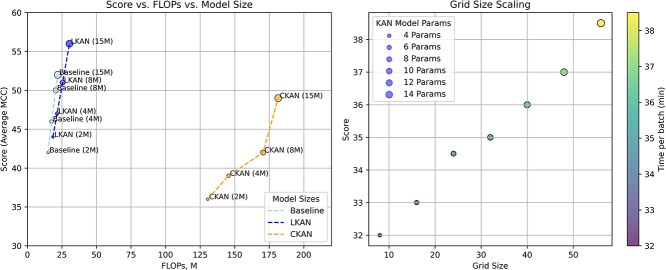
Scaling of models. A. Comparison of the tested models of various sizes, evaluated on different collections of datasets. B. Linear KAN grid size scaling on model’s parameters and training time on GUE+ dataset.

One of the reasons why CKAN did not perform equally well with LKAN with the same number of parameters is that more experiments are needed to determine the best place for CKAN in the architecture (Fig. 1). In our set of experiments, we followed the best practices presented in [27, 28], suggesting the rationale for putting CKAN into a mapping block. In computer vision, experiments with parameter-lighter models [27] showed that placing CKAN may enhance the performance by 0.8%–1.5%. We did not perform the placement experiments due to the lack of resources but we think there is a positive trend for larger CKAN sizes as indicated in the transition from 8M to 15M (Fig. 4). With more advanced implementation techniques (replacing B-splines with GPU parallelizable functions [36]) and training techniques (parallelizable Batch Ensembling [60]) it could be possible to scale CKANs further. Yet, in our work, we conclude that for this moment, LKAN incorporated into LegNet-like architecture [54] may be used as an improvement for downstream classification.

**Figure 5:**
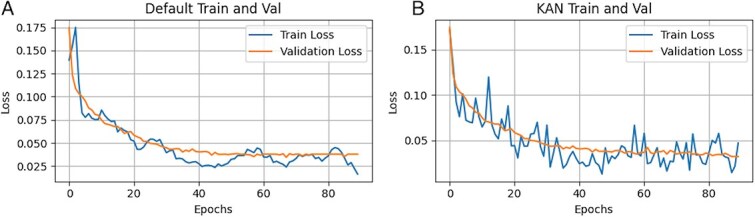
Loss functions of generative models. A. Baseline Unet incorporating EMA. B. Unet with MLP replaced by LKAN and incorporating EMA.

**Figure 6:**
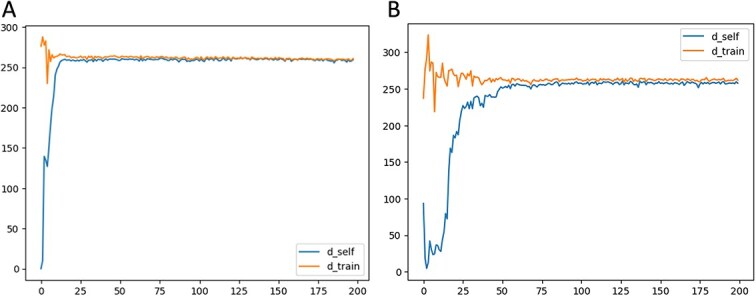
Edit distances for Wasserstein GANs. A. WGAN with KAN linear layer (see Fig. 3B). B. Vanilla GAN (see Fig. 3A).

**Figure 7:**
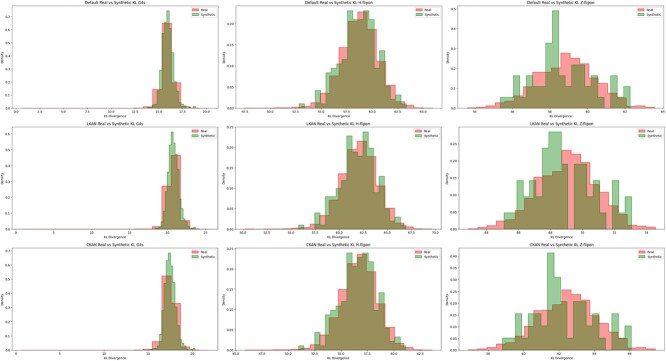
Distributions of Kullback–Leibler divergence for real and synthetic data produced by Baseline Unet (Default) and Unet with LKAN on flipon benchmark datasets.

**Figure 8:**
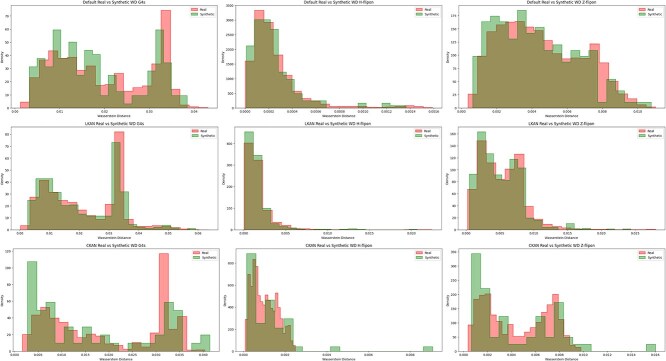
Distributions of Wasserstein Distance for real and synthetic data produced by Baseline Unet (Default) and Unet with LKAN on flipon benchmark datasets.

In different domains, such as computer vision and natural language processing, it is not yet clear if KANs significantly outperform MLPs as it is a topic of active discussions [28, 32, 35, 61]. Our results are preliminary since we have not tested models with large grid size parameters and did not test other types of KANs. Recent advancements in the KAN field inlcude RNN-based and Transformer-based KANs. Temporal Kolmogorov–Arnold Networks using Long Short-Term Memory mechanism to leverage time dependency in KANs were proposed in [33]. Kolmogorov–Arnold Transformer (KAT) architecture is based on Vision Transformer [62] and utilizes rational polynomial functions to replace splines [29]. KATs were applied to computer vision tasks and, to our knowledge, have not been yet adapted for natural language processing, making it difficult for testing on genomic tasks.

An additional long training (500 epochs) experiment with H3 dataset revealed that training of LKAN is 2.7 times longer than the Baseline. Also we do not observe the trend that LKAN can generalize faster than the Baseline given the same amount of training steps. We consider the different number of epochs for different datasets as they vary in sizes and complexity. In addition, we observe that LKAN can overfit with large grid size parameter (Supplementary Figure 1) after a long training.

To outline the computational difference between standard convolutions and Kolmogorov–Arnold convolutions we conducted an additional experiment with comparison of one CKAN layer with one convolutional layer (Supplementary Table 18). The results showed that for small batch of shape [1, 4, 500], it takes 103 ms and 6000 FLOPs for conv1d layer from pytorch versus 2 s (x19) and 62 000 FLOPs (x10) for CKAN. Authors in [27] report that in the computer vision domain for ImageNet datasets their implementation of KAN Conv training 10x longer than for vanilla CNN. MLPs were compared with KANs on tabular data [63] and they observe the same computational behavior: KANs require significantly more (x15) FLOPs than MLPs—see Fig. 2 in [63]. Replacement of B-Splines by Radial Basis Function, which is GPU parallelizable, may significantly enhance the training and tackle CKAN scalability [36].

Current implementations of LKAN lack the same interpretability as original KAN [24] offers: an ability to plot and prune the latest layers and suggest symbolic expression for outlying dependencies between variables. Potentially, this could bring even more information about inner structure of a biological task of interest.

Research in the KAN field is evolving rapidly as more and more studies emerge and suggest various technical improvements to address challenges related to KAN usage. The future directions may involve training a Transformer-based models with KANs to handle the task of studying latent representations of a language model’s hidden states—embeddings. It was shown [21, 64] that visualization of language model embeddings could enhance biological understanding of model’s utility. Of interest is to test more frameworks as state-space models [22], and transformers with GPT-like architecture [65]. In addition, there are multiple advancements in sequence generative modeling like Discrete Diffusion for DNA [66]. Despite our first positive experiments with linear and convolutional KANs, unleashing the potential of KAN for the entire spectrum of genomic tasks requires further extensive research.

## Supplementary Material

KAN_DNA_Supplementary_110225_bbaf129
